# Concurrent pancreatic ductal adenocarcinoma and poorly differentiated neuroendocrine carcinoma: A case report and review of the literature

**DOI:** 10.1016/j.ijscr.2025.111320

**Published:** 2025-04-28

**Authors:** Alireza Negahi, Ali Zare-Mirzaie, Hossein Negahban, Sepideh Soleymani, Ali Jaliliyan, Shahram Agah

**Affiliations:** a Department of Surgery, Breast Health & Cancer Research Center, Iran University of Medical Sciences, Tehran, Iran; b Department of Pathology, School of Medicine, Rasool-E Akram Hospital, Iran University of Medical Sciences, Tehran, Iran; c Department of Surgery, Surgery Research Center, School of Medicine, Rasool-E Akram Hospital, Iran University of Medical Sciences, Tehran, Iran; d Department of Internal Medicine & Gastroenterology, School of Medicine, Iran University of Medical Sciences, Tehran, Iran

**Keywords:** Pancreatic ductal adenocarcinoma, Pancreatic neuroendocrine carcinoma, Whipple procedure, Histopathology, Pancreatic cancer, Case report

## Abstract

**Introduction:**

Concurrent pancreatic ductal adenocarcinoma (PDAC) and poorly differentiated neuroendocrine carcinoma (NEC) is a rare condition. This simultaneous occurrence poses significant diagnostic and therapeutic challenges due to the unique characteristics and treatment protocols of each cancer. An accurate diagnosis is crucial to optimizing treatment outcomes and prognosis.

**Presentation of case:**

We present a case of a 55-year-old male with type 2 diabetes and psoriatic arthritis, referred for an elevated serum CA 19–9 level found during a check-up. Imaging studies, including CT and EUS, revealed a 6 cm mass in the pancreatic head. EUS-guided biopsy confirmed PDAC. After a Whipple procedure, pathology showed concurrent poorly differentiated NEC with a 30 % neuroendocrine component. After surgery, the patient received gemcitabine-based chemotherapy and was disease-free at six months post-surgery.

**Discussion:**

This case illustrates the diagnostic intricacy of simultaneous PDAC and poorly differentiated NEC. Effective management in such scenarios benefits from a collaborative approach among surgeons, oncologists, and pathologists. Due to the limited number of documented cases, there is insufficient evidence to inform the best treatment strategies, particularly concerning the most effective chemotherapy options. This case adds to the growing body of literature on rare concurrent pancreatic tumors and highlights the need for further research to enhance understanding and develop comprehensive clinical guidelines.

**Conclusion:**

The combination of PDAC and poorly differentiated NEC poses unique diagnostic and treatment challenges. This case underscores the importance of a multidisciplinary approach and calls for further research to develop evidence-based management protocols for these rare malignancies.

## Highlights

• This report discusses a rare case of concurrent PDAC and NEC.
• A multidisciplinary approach is needed for managing concurrent pancreatic tumors.
• The patient had a Whipple procedure and was disease-free after six months.
• The report urges research for guidelines on rare dual pancreatic cancers.

## 1 Introduction

Pancreatic cancer represents a significant global health challenge, ranking among the most lethal malignancies worldwide [1,2]. Despite its relatively low incidence, it is a leading cause of cancer-related mortality, with poor overall survival rates with a 5-year survival rate of approximately 11–12 % [3,4]. Two primary histological subtypes exist: pancreatic ductal adenocarcinoma (PDAC), characterized by aggressive behavior and poor prognosis, and pancreatic neuroendocrine tumors (PNETs), which exhibit more benign clinical behavior. These distinct entities differ substantially in pathophysiology, clinical manifestation, molecular characteristics, and patient outcomes.

PDAC predominantly develops through progressive genetic mutations, with key risk factors including smoking, chronic pancreatitis, diabetes, and inherited genetic syndromes. Typically asymptomatic until advanced stages, and often presents with nonspecific symptoms like weight loss, jaundice, and abdominal pain [5]. Conversely, PNETs arise from hormone-producing cells, ranging from well-differentiated, indolent lesions to rare, aggressive, poorly differentiated neuroendocrine carcinomas (NECs), which exhibit markedly different clinical behaviors and molecular characteristics [6].

The World Health Organization (WHO) defines concurrent tumors as separate malignant neoplasms located in the same organ or anatomical site, consisting of at least two different malignant components without any mixed or transitional regions [7]. The incidence of PNET in the United States is estimated to be around 0.27 to 1 case per 100,000 people annually, with a very low likelihood of concurrent occurrence with pancreatic ductal adenocarcinoma; therefore, the simultaneous occurrence of PDAC and PNETs represents an infrequent clinical scenario [8,9]. This case report and literature review present an instance of concurrent PDAC and poorly differentiated NEC, aiming to expand our understanding of rare concurrent pancreatic malignancies.

## 2 Case presentation

### 2.1 Clinical presentation and medical history

The case report has been reported in line with the SCARE criteria [10]. A 55-year-old white man was admitted in July 2024 to evaluate the elevated serum CA 19–9 level observed in an incidental check-up. The patient had a history of type 2 diabetes mellitus controlled by oral Metformin and Sitagliptin, and psoriatic arthritis treated with oral corticosteroids. There were no other notable past medical or family histories. The patient had no abdominal pain, weight loss, jaundice, nausea, or vomiting. The abdominal examination revealed no tenderness or rebound tenderness.

### 2.2 Diagnostic evaluations

**Fig. 1:**
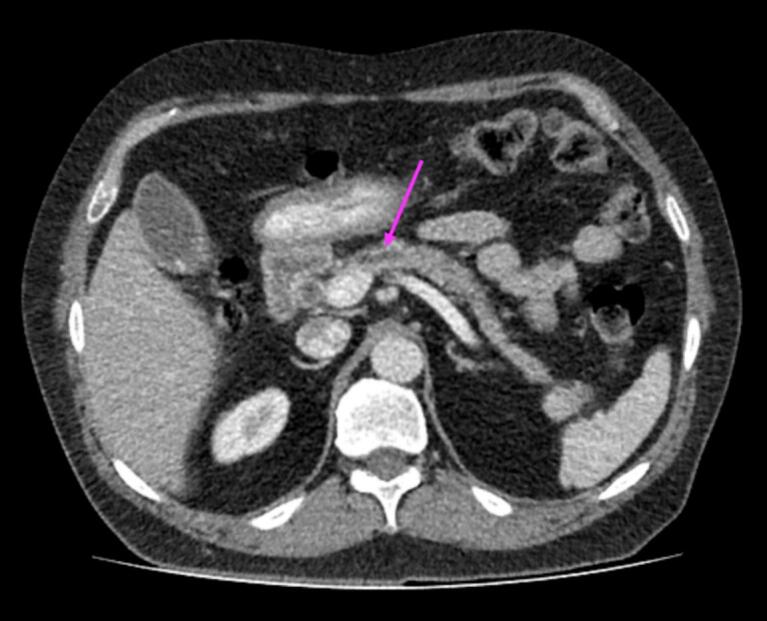
Irregular-shaped hypoechoic lesion at the head of the pancreas in contrast-enhanced Abdominal CT scan, Portal phase.

Laboratory examination revealed elevated liver enzymes with an obstructive pattern (Aspartate Aminotransferase (AST): 130 IU/L, Reference value: 5–40; Alanine Transaminase (ALT): 196 IU/L, Reference value: 5–40; Alkaline Phosphatase (ALP): 990 IU/L, Reference value: 64–306; Direct Bilirubin: 0.3, Reference Value: 0–0.4; Total Bilirubin: 0.8, Reference Value: 0.2–1) and an increased CA19–9 level (250 IU/mL, Reference value: 0–37 IU/mL). Triple-phase computed tomography (CT) examination detected a 6 cm hypoechoic mass at the head of the pancreas with no Main Pancreatic Duct (MPD) dilation (Fig. 1).

The transabdominal sonographic evaluation revealed a hypoechoic, ill-defined mass in the head of the pancreas, measuring approximately 6 cm (longest diagonal) and no common bile duct (CBD) dilation, suggestive of performing an Endoscopic Ultrasonography (EUS) with a needle biopsy. The mass was observable in EUS with no MPD dilation. EUS-guided needle biopsy results indicated high cellularity with a dense population of epithelial cells with poorly cohesive, disorganized architecture, frequent mitotic figures, necrotic debris, and other specific immunohistochemistry characteristics highly suggestive of PDAC.

### 2.3 Surgery and pathologic evaluation

A Whipple procedure was performed. No peritoneal or liver metastasis was observed. The Superior Mesenteric Artery (SMA), Superior Mesenteric Vein (SMV), and Portal Vein (PV) were not invaded by the tumor. The post-operative period was uneventful. The resected specimen was examined macroscopically, and a 6 × 4 × 3 cm ill-defined mass was found at the head of the pancreas. Microscopic evaluation revealed a mixed, well-differentiated PDAC concurrent with a poorly differentiated NEC, with a 30 % neuroendocrine component and a 10 % Ki-67 proliferation index. The tumor had invaded the Ampulla of Vater and the duodenal wall. The retroperitoneal, proximal and distal pancreatic, and CBD margins were free of tumoral tissue. Lymphovascular invasion was not identified, but perineural invasion was present. Four regional lymph nodes were examined in the resected specimen, with two nodes involved by PDAC and two nodes not invaded. A pathological stage of T3N1Mx was reported.

**Fig. 2:**
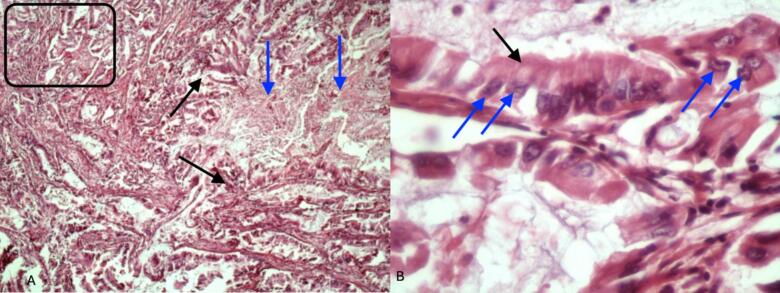
PDAC: A (left - ×100). Glandular structures infiltrating the pancreatic stroma (Black arrows), fibrotic stroma surrounding the glandular elements (blue arrows), pleomorphism: glandular cells have varied size and shape (black rectangle); B (right - ×1000). Abundant eosinophilic cytoplasm (black arrow), prominent nucleoli (blue arrows), and irregular nuclear contour (blue arrows).

The tumor's PDAC component consisted of mucinous, glandular structures with moderate pleomorphism. The tumor cells had abundant eosinophilic cytoplasm and prominent nucleoli. The glandular architecture was well-formed, though somewhat irregular, with areas of cribriform architecture and occasional intraluminal necrosis. The stroma was desmoplastic, with abundant fibrous tissue surrounding the tumor cells, accompanied by a lymphocytic inflammatory infiltrate. The nuclei in the PDAC component were pleomorphic with prominent nucleoli and mild nuclear atypia (Fig. 2).

**Fig. 3:**
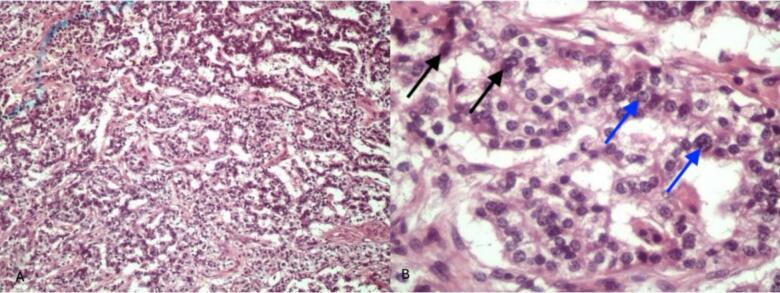
NEC: A (left - ×100). Highly cellular sheets of small—to medium-sized cells with scant cytoplasm and irregular, hyperchromatic nuclei (×100); B (right - ×1000). Hyperchromatic nuclei (Black arrows), irregular nuclear contour, and the salt-and-pepper appearance (Blue arrows).

**Fig. 4:**
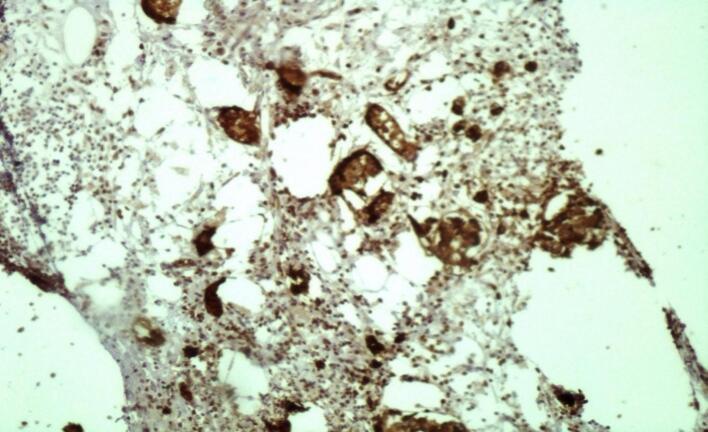
Immunohistochemical synaptophysin examination, positive endocrine cells.

The tumor's neuroendocrine component consisted of highly cellular sheets of small—to medium-sized cells with scant cytoplasm and irregular, hyperchromatic nuclei. The nuclear contour was markedly irregular, and the chromatin, characteristic of neuroendocrine tumors, had a salt-and-pepper appearance. High mitotic activity, with numerous mitotic figures (>20 mitoses per 10 high-power fields) and areas of necrosis, was observed, indicative of the tumor's aggressive nature (Fig. 3). Synaptophysin+ endocrine cells were present at the immunohistochemistry examination (Fig. 4).

### 2.4 Patient follow-up

After the surgery, the patient received gemcitabine-based chemotherapy, with close monitoring of renal and liver function, as well as serum glucose levels. Periodic serum Ca 19–9 examinations and abdominal CT imaging were done every three months for the patient. The patient did not have any signs of relapse six months after the surgery.

## 3 Discussion

This case report describes the rare presentation of concurrent PDAC and poorly differentiated NEC in a single patient. Such co-occurrence poses unique diagnostic, therapeutic, and prognostic challenges.

Surgical resection is a key treatment for both tumor types [11,12], yet the extent of excision can vary. The Whipple procedure is typically preferred for PDAC, which arises from the head or body of the pancreas. At the same time, a distal or total pancreatectomy might be utilized for other invasive neuroendocrine tumors originating from the distal body or the tail. This distinction is crucial because the two surgical methods present different complication risks. For instance, the Whipple procedure carries a relatively high likelihood of serious complications, such as pancreatic fistula, which occurs quite frequently [13]. Conversely, the distal or total pancreatectomy, which maintains the duodenum, generally has a lower chance of these severe complications. The clinical implications of choosing the appropriate surgical approach are particularly important in this case, where the patient's unique dual malignancies necessitate careful consideration of both tumor types' characteristics to minimize surgical risk and improve postoperative outcomes.

Another critical consideration in such tumors is the challenge of selecting the appropriate adjuvant chemotherapy regimen after surgery. Chemotherapy for pancreatic adenocarcinoma primarily relies on combination regimens such as FOLFIRINOX [14] and gemcitabine plus nab-paclitaxel [15], both of which have demonstrated significant improvements in overall survival. Emerging targeted and immunotherapies are also under investigation [16]. In contrast, high-grade pancreatic neuroendocrine carcinomas (NECs) are treated with cisplatin-based chemotherapy, though responses are often short-lived. Alkylating and platinum-based regimens show greater efficacy in grade 3 neuroendocrine tumors [17,18]. The key difference lies in treatment durability—while adenocarcinoma therapies focus on prolonging survival, NEC treatments face challenges with rapid progression and resistance, necessitating more effective therapeutic strategies. However, there is still insufficient evidence regarding the optimal chemotherapy regimen that can minimize the risk of recurrence in patients concurrently diagnosed with both tumor types, specifically invasive tumor types like poorly differentiated NEC.

Another important aspect of this case is the history of autoimmune-related diseases. The development of autoimmunity and its potential link to cancer involves complex mechanisms that include immune dysregulation and chronic inflammation. Autoimmunity occurs when the immune system fails to maintain tolerance to self-antigens, leading to the activation of autoreactive immune cells and chronic tissue damage [19,20]. This dysregulation can create an inflammatory environment conducive to tumorigenesis, where specific autoantibodies correlate with increased cancer risk [21]. While various studies support the connection between autoimmunity and cancer, the exact mechanisms remain under investigation, with ongoing research needed to clarify these relationships and their implications for treatment and prevention strategies.

The genetic aspects of pancreatic cancer are another matter of importance. While both PDAC and NEC can exhibit TP53 mutations, PDAC is primarily defined by KRAS, CDKN2A, and SMAD4 mutations, whereas NEC is characterized by ATRX, DAXX, and MEN1 mutations [[22], [23], [24]]. This genetic distinction may explain the concurrent occurrence of such malignancies.

The literature on concurrent pancreatic tumors is limited, with only a few case reports and series available. Clinical presentation varies significantly. Abdominal pain was a common symptom in some cases [25,26], while in others, including our case, the tumor was an incidental finding [27]. Type 2 diabetes was present in several instances [26,28], supporting the potential link between pancreatic tumor pathology and diabetes, a feature also seen in our patient.

The location of the tumors varied across cases, with some in the pancreatic head necessitating a Whipple procedure [27] and others in the body or tail managed with distal pancreatectomy [25,26,28,29]. Tumor sizes ranged, with our case having one of the largest tumors reported in the literature. Larger tumors are often associated with a poorer prognosis.

CA 19–9, a biomarker for pancreatic cancer, was elevated in several cases [25,26,28,29], including our own. This elevation supported the clinical suspicion of malignancy. Interestingly, elevated CA 19–9 was not observed in some cases despite a pancreatic tumor [27]. Therefore, normal serum Ca 19–9 levels may not be useful in ruling out pancreatic tumors. Surgical intervention was the primary treatment strategy. Cases with tumors in the pancreatic head, like ours, required a Whipple procedure, while tumors in the body or tail were managed with distal pancreatectomy. Adjuvant chemotherapy was administered in most cases, consistent with guidelines for PDAC with lymph node involvement. Our approach also included adjuvant chemotherapy, particularly given the aggressive nature of NEC and lymph node involvement.

Another matter of importance in the context of concurrent pancreatic tumors is collision tumors. Collision tumors consist of two distinct neoplasms that develop independently and remain separate [30]. In our literature review, we found two cases of collision tumors [28,29], but our case does not fit this definition. The exact pathogenesis of these collision neoplasms also remains unclear. Further research is needed to clarify their development and clinical significance.

Patient outcomes varied significantly. Developing a postoperative pancreatic fistula, tumor recurrence, and death were documented in one of the reported cases [27]. On the other hand, some cases did not face any complications after the surgery and during the follow-up process.

Evidence regarding various aspects of concurrent pancreatic tumors is scarce, particularly concerning the best adjuvant chemotherapy regimen, long-term survival outcomes, and the underlying mechanisms of the phenomenon. Existing treatment guidelines are based on cases involving single tumors, and there are no established protocols for addressing this dual malignancy. Additional research is essential to identify the most effective treatment approaches and enhance patient outcomes.

## 4 Conclusion

The concurrent occurrence of PDAC and poorly differentiated NEC is exceedingly rare and diagnostically challenging. This case emphasizes the importance of comprehensive pathological examination to identify concurrent malignancies, as treatment and prognosis differ significantly between PDAC and NEC. This case highlights the need for a more tailored adjuvant chemotherapy approach, as standard PDAC regimens may be insufficient to address the different nature of poorly differentiated NEC. Also, the immunologic disturbances presented by this patient probably favored the emergence of this concomitant malignancy. Further studies are necessary to establish standardized guidelines for the diagnosis, treatment, and follow-up of patients with such rare dual pancreatic malignancies.

## CRediT authorship contribution statement

**Negahi A:** Study conception, Data collection, Critical revision of the manuscript, Supervision
**Zare-Mirzaei A:** Data interpretation, Critical revision of the manuscript
**Negahban H:** Data collection, Critical revision of the manuscript
**Soleymani S:** Data collection, Drafting the manuscript
**Jaliliyan A:** Study conception, Drafting the manuscript
**Agah H:** Data collection, Data interpretation, Critical revision of the manuscript, Supervision.

## Patient consent

Written informed consent was obtained from the patient to publish this case report and accompanying images. A copy of the written consent is available for review by the Editor-in-Chief of this journal on request.

## Ethical approval

Case report studies are exempt from ethical approval in your institution if no personal information about the patient is included in the article.

## Guarantor

Shahram Agah.

## Research registration number

The Case Report is not considered as “First in Man.”

## Funding

This research was conducted without any external financial support.

## Declaration of competing interest

The authors declare that they have no conflicts of interest regarding the publication of this research.
